# *MDM2* SNP309 polymorphism is associated with lung cancer risk in women: A meta-analysis using METAGEN

**DOI:** 10.3892/etm.2012.640

**Published:** 2012-07-18

**Authors:** WENWU HE, JIANXIONG LONG, LEI XIAN, FENG PANG, LI SU, SHIXIU WEI, BO WEI, YANLING HU

**Affiliations:** 1 Department of Cardiothoracic Surgery, First Affiliated Hospital, Guangxi Medical University, Nanning, Guangxi;; 2 School of Public Health; 3 Fourth Grade of Clinical Medicine and; 4 Medical Research Center, Guangxi Medical University, Nanning, Guangxi, P.R. China

**Keywords:** murine double minute 2, SNP309, polymorphism, lung cancer, meta-analysis

## Abstract

Lung cancer is the most common diagnosed malignancy and the leading cause of cancer-related mortality worldwide. Murine double minute 2 (*MDM2)* SNP309 polymorphisms have been reported to influence the risk of lung cancer. However, the published studies together with four subsequent meta-analyses have yielded contradictory results. To examine this inconsistency, we conducted a meta-analysis of 6,696 lung cancer cases and 7,972 controls from eight published case-control studies using METAGEN. Odds ratios (ORs) with 95% confidence intervals (CIs) were calculated with STATA software and used to assess the strength of the association. In the overall analysis, a significant association between *MDM2* SNP309 polymorphism and lung cancer risk was observed (OR, 1.143; 95% CI, 1.047–1.247). Moreover, stratified by ethnicity, a significant association was found in Asians (OR, 1.260; 95% CI, 1.111–1.429), but not in Europeans. Subgroup analysis of gender, histology and smoking status suggested that the *MDM2* SNP309 genotype was associated with increased lung cancer risk in women (OR, 1.282; 95% CI, 1.062–1.548) and never smokers (OR, 1.328; 95% CI, 1.119–1.575). No statistically significant association was observed in males and ever smoking population, and no association was found in subgroup analysis based on histology. In conclusion, the association between *MDM2* SNP309 and lung cancer was statistically significant, particularly in Asians, women and never smoking population.

## Introduction

Lung cancer is the most common malignancy diagnosed in males, and the fourth most common diagnosed malignancy in females worldwide. Moreover, it is the leading cause of cancer-related mortality in males and the second leading cause of cancer-related death in females. Based on the GLOBOCAN 2008 estimates, lung cancer accounts for 13% (1.6 million) of the total cases and 18% (1.4 million) of deaths in 2008 (1). Lung cancer is a complex, multistage and multifactor disease. Smoking and environmental or occupational exposure may increase the risk of lung cancer; inherited polymorphisms including carcinogen metabolism (2), DNA damage and repair capacity (3), cell apoptosis (4) and cell-cycle control (5) also play important roles in determining interindividual variations in lung cancer susceptibility.

The murine double minute 2 (*MDM2*) gene located on chromosome 12q13–14 encodes an important negative regulator for the p53 tumor suppressor, resulting in its ubiquitination by direct binding to p53 (6). Apart from this critical function, *MDM2* overexpression may inhibit DNA repair independent of p53 (7,8). The carcinogenesis of *MDM2* overexpression has been observed in various human tumors including lung (9–11), breast (12), bladder (13), gastric (14) and colorectal cancers (15).

An SNP (SNP309, T to G change at nucleotide 309) in the first intron of the *MDM2* gene was identified and was found to be associated with an increased affinity for binding stimulatory protein SP1 to the promoter region, resulting in increased *MDM2* gene transcription and attenuation of the p53 pathway (16). Subsequently, several molecular epidemiological studies were conducted to assess the association between *MDM2* SNP309 and lung cancer risk in Asians (10,17–19) Caucasians (9,20–22) and African-Americans (11). However, the results were not always consistent, possibly attributable to the fact that the majority of studies featured only a small number of samples or the association between the *MDM2* SNP309 polymorphism and lung cancer risk did not achieve statistical significance. Two meta-analyses published in 2009 exploring the *MDM2* SNP309 polymorphism and lung cancer risk yielded contradictory results (23,24) and other two meta-analyses investigating *MDM2* SNP309 and tumor susceptibility published in 2011 also showed inconsistent results (25,26). To resolve these inconsistencies, we conducted the present meta-analysis of available case-control studies investigating the association between *MDM2* SNP309 and lung cancer risk using METAGEN (27). In addition to performing subgroup analyses based on ethnicity and smoking status as the previous meta-analyses (23,24), we conducted subgroup analyses involving gender and histology using the most appropriate genetic model.

## Materials and methods

### Search strategy

Pubmed, Embase and HuGENet databases were searched using the following terms or combinations: ‘murine double minute 2’, ‘SNP309’, ‘*MDM2*’, ‘polymorphism’ and ‘lung cancer’. The search was conducted until February 2012, and was limited to English language studies. To obtain additional potentially eligible studies we also reviewed the reference lists of the original studies identified, as well as reviews and previous meta-analyses.

### Inclusion and exclusion criteria

The eligible studies assessed in our meta-analysis had to achieve all of the following criteria: i) original studies concerning *MDM2* SNP309 polymorphism and lung cancer risk, ii) studies containing useful genotype distribution information, iii) case-control or cohort studies and iv) studies with a control population without prior or current severe respiratory disease and malignant disease. The exclusion criteria were: i) studies analyzing the same patient population (the most recent or complete study was chosen) and ii) case-only studies, which were studies without a healthy control group.

### Data extraction

From each eligible study, we extracted data consisting of the first author, year of publication, ethnicity of the population, the genotype frequency of the cases and controls, diagnostic criteria and genotyping methods. For studies including the the frequency of genotypes of the cases and controls according to different subgroups for gender, histology and smoking status, data were separately extracted for each subgroup whenever possible.

### Statistical analysis

Hardy-Weinberg equilibrium (HWE) for the genotype distributions in the controls of each study was assessed using Pearson’s Square (P≥0.05) (28). Cochran’s Q test (29) and the inconsistency index (*I^2^*) (30) were next calculated for assessment of heterogeneity. We considered P<0.05 and I^2^>50% to indicate significant heterogeneity (31,32). In this case, we checked the data and statistical methods again, and conducted sensitivity analysis or subgroup analysis to analyze the reasons for heterogeneity. If significant heterogeneity was present, the random-effects model was used, or else, the fixed-effect model was chosen. Next, logistic regression analysis was performed to examine the association between the *MDM2* SNP309 T>G polymorphism and lung cancer risk. Three genetic models: dominant (GG/TG vs. TT), recessive (GG vs. TG/TT) and co-dominant (GG vs. TG and TG vs. TT) were used to evaluated the risk. The methodology used to determine the most appropriate genetic model was reported by Bagos and Nikolopoulos (27). Two parameters θ_2_ and θ_3_ were calculated using the formula: log it (π_ij_) = α_i_ + θ_2_ z_i2_+θ_3_ z_i3_ and OR_AB/AA_= exp(θ_2_), OR_BB/AA_= exp(θ_2_); where α_i_ are indicators of the study-specific fixed-effects, and θ_2_ and θ_3_ are dummy variables of genotypes AB and BB. The appropriate genetic model was identified using the following criteria (33):

- no association: θ_2_=θ_3_ (OR_AB/AA_ = OR_BB/AA_ = 1)
- dominant model: θ_2_≠0, θ_3_≠0 and θ_2_=θ_3_ (OR_AB/AA_ = OR_BB/AA_≠1)
- recessive model: θ_2_=0 (OR_AB/AA_ = 1) and θ_3_≠0 (OR_BB/AA_ ≠ 1)
- co-dominant model: θ_2_≠0, θ_3_≠0 and 2θ_2_=θ3 (OR^2^_AB/AA_ = OR_BB/AA_)

Finally, once the most approtriate genetic model was identified, it was used to again pool the results for both logistic regression analysis and heterogeneity assessment. Subgroup analysis also abided by this procedure.

Publication bias was assessed using Egger’s tests and Begg’s funnel plot (34). The statistical analysis was performed using METAGEN (http://bioinformatics.biol.uoa.gr/~pbagos/metagen/) and STATA version 11.1 (Stata Corporation, USA). All P-values were two-sided.

## Results

### Study inclusion and characteristics

According to the inclusion and exclusion criteria, we extensively searched the Pubmed, Embase and HuGENet databases. There were 14 original studies relevant to the associated of *MDM2* SNP309 polymorphism and lung cancer risk retrieved. We reviewed all full-text studies intensively and excluded four studies without a control group (35–38). The study of Jun *et al* (39) was excluded since it included the same case and control population as Park *et al* (17). Finally, we excluded the study of Chua *et al* (19) since this study focused on the relevance between *MDM2* SNP309 and lung cancer risk among non-smoking women, while the other studies included no gender and smoking state restriction. Thus, eight case-control studies (6,696 lung cancer cases and 7,972 controls) concerning the associated of the *MDM2* SNP309 polymorphism and lung cancer risk were included in this meta-analysis (9–11,17,18,20–22). The study of Pine *et al* (11) reported a population consisting of Caucasians and African-Americans, thus we recorded data on *MDM2* SNP309 genotype distributions, respectively.

All of the eligible studies were consistent with Hardy-Weinberg equilibrium. The characteristics of the studies are summarized in Table I, and frequencies of the *MDM2* 309T>G polymorphism in the different populations are documented in Table II. Among these studies, some provided information on *MDM2* SNP309 genotype frequencies in different subgroups based on gender, histology and smoking status (Table III).

### Meta-analysis results

Table IV shows the detailed results of the heterogeneity test, assessment of the most appropriate genetic model, and the association between *MDM2* SNP309 T>G polymorphism and lung cancer risk evaluated using odds ratios (ORs) with 95% confidence intervals (CIs). In the overall analysis, using the fixed-effect model, significant associations between the *MDM2* SNP309 polymorphism and lung cancer risk were observed in the recessive model (OR, 1.143; 95% CI, 1.047–1.247). Moreover, following the stratification of studies according to ethnicity, a statistically significant association was noted in Asian (OR, 1.260; 95% CI, 1.111–1.429) while not in European population. In the subgroup analysis based on gender, we found that the *MDM2* SNP309 GG genotype conferred a statistically significant increased risk of lung cancer development in females (OR, 1.282, 95%; CI, 1.062–1.548). However, we did not observe a similar result in males. In the subgroup analysis based on smoking status, a statistically significant association was observed in never smokers (OR, 1.328; 95% CI, 1.119–1.575) but not in the ever smoking group. However, there was no evidence of a statistically significant association between the *MDM2* SNP309 polymorphism and lung cancer risk in the subgroup analysis based on histology.

### Publication bias

We performed Egger’s test to access the publication bias. There was no indication that the shapes of the funnel plots exhibited obvious asymmetry, indicating any obvious evidence of publication bias in this meta-analysis.

## Discussion

Previous meta-analyses elucidating the link between the *MDM2* SNP309 polymorphism and lung cancer risk have provided discrepant results. Gui *et al* (24) found that the *MDM2* 309G allele is a low-penetrant risk factor for developing lung cancer in Asians, yet Bai *et al* (23) conducted a meta-analysis involved in the same research and found that the *MDM2* SNP309 GG genotype enhanced the risk of lung cancer development in never smokers with statistical significance, but no statistically significant was noted in Asian and European individuals. The main difference between these two meta-analyses was the genetic model selection. Hence, we conducted the present meta-analysis, one study more than these two meta-analyses (23,24) using METAGEN. Apart from finding that the *MDM2* SNP309 polymorphism increases the risk of lung cancer development with a statistical significance, particularly in never smoking and Asian populations, we explored the relationships between the *MDM2* SNP309 polymorphism and the risk of lung cancer based on subgroups of different gender and histology using the most appropriate genetic model. We detected a statistically significant association between the *MDM2* SNP309 polymorphism and the risk of lung cancer in females.

According to the results of this meta-analysis, our main finding was that the *MDM2* SNP309 polymorphism was statistically significantly associated with the risk of lung cancer in females but not in males. It appears that the results of the subgroup analysis according to females and males was due to smoking and no smoking status as more men smoke than women, and it has been suggested that *MDM2* SNP309 increases the risk of lung cancer in never smokers (21,23,40). Yet, this assumption counteracts with the traditional view that tobacco smoking causes lung cancer. We prefer to believe that the estrogen receptor α (ERα) regulates *MDM2* SNP309 expression and leads to an increased risk of lung cancer in females. This indicates that men and women have approximately the same prevalence of lung cancer although smoking status tends to be higher among men compared to women.

The *MDM2* SNP309 G allele frequency was found to be a risk factor in Asians. This result is consistent with previous studies (17,18). The possible cancerogenic mechanism of the *MDM2* SNP309 polymorphism was previously documented. However, the reason for the differential effects of the *MDM2* polymorphism according to ethnicity is unclear. The differences in environment and genetic backgrounds may influence the association between the *MDM2* SNP309 polymorphism and risk for lung cancer. Here, we must mention that, following subgroup analysis of Asian individuals, the result of our meta-analysis exhibited dissimilarity with Bai *et al* (23), which included the same studies conducted in Asians (10,17,18). The main reason is the selection of the genetic model. We identified the most appropriate genetic model using METAGEN as described by Bagos and Nikolopoulos (27) and this method allowed for heterogeneity between studies, thus our results should be more accurate.

Heterogeneity is inevitable in a meta-analysis (41,42). We have to admit heterogeneity also existed between all the included studies in our meta-analysis. Sources of heterogeneity may come from various channels. First, studies included in this meta-analysis were distributed according to different ethnicity and environment. In addition, a different methodology including the source of controls, diagnostic criteria and genotyping methods may lead to heterogeneity.

Other possible limitations should be taken into account which contributed to the low statistical power of this meta-analysis. For instance, we only selected studies published in the electronic edition of the databases in English language and ignored the studies published in other languages, in paper version or not published at all. Publication bias may exist although Begg’s funnel plot and Egger’s test did not detect bias. Additionally, not all the included studies contained the complete data needed particularly for the subgroup analysis, thus we only analyzed the obtained data.

In conclusion, the current meta-analysis demonstrates that the *MDM2* SNP309 GG genotype may increase the risk of lung cancer particularly in Asians, females and never smoking population. Considering the limitations of this meta-analysis, further studies with large sample sizes, using well-designed and more accurate methods of genotyping are warranted to confirm the association between *MDM2* SNP309 and lung cancer risk.

## Figures and Tables

**Table I:** Characteristics of all eligible studies included in this meta-analysis.

			Sample size		Diagnostic criteria				
Study (ref.)	Year	Ethnicity	Cases	Controls	Cases	Controls	Genotyping methods	Source of controls	P-value for HWE
Hu *et al* (10)	2006	Asian	717	1,083	Patients with histopathologically confirmed lung cancer.	Cancer-free controls were frequency-matched to the cases regardingage (65 years), gender and residential area (urban or countryside).	PIRA-PCR	Population	0.832
Li *et al* (9)	2006	Caucasian	1,026	1,145	Patients had newly diagnosed, histopathologically confirmed, previously untreated (i.e. by radiotherapy or chemotherapy) lung cancer without restrictions on age, gender, stage or histology.	Controls were frequency-matched to the cases regarding age (±5 years), gender, ethnicity and smoking status (i.e. ever and never). Patients previously receiving radiotherapy or chemotherapy or recent (in last 6 months) blood transfusions or with previous cancer were excluded.	PIRA-PCR	Hospital	0.101
Lind *et al* (20)	2006	Caucasian	341	412	Cases were newly diagnosed lung cancer patients treated by surgery. Tumor histology was confirmed by an experienced pathologist, and only NSCLC cases were included in the study.	Controls had a mean age of 59 years, smoked >5 cigarettes/day, were current smokers or quit smoking for <5 years prior to study.	TaqMan	Population	0.059
Park *et al* (17)	2006	Asian	582	582	Patients were newly diagnosed with primary lung cancer, and patients with a prior history of cancers were excluded.	Control subjects were randomly selected from a pool of healthy volunteers who visited the general health check-upcenter. They were frequency-matched (1:1) to the cases based on gender and age (±5 years).	PCR-RFLP	Population	0.438
Pine *et al* (11)	2006	Caucasian	371	421	All cases were histologically confirmed non-small cell primary tumors of the lungs. Patients were currently not taking antibiotics or steroid medications and had not previously undergone chemotherapy or radiation therapy (43).	Hospital controls were cancer-free patients recruited from the same hospital as the cases and were frequency-matched to the cases by gender and ethnicity. Population controls were recruited from Baltimore City and the same counties to match cases by age, gender and ethnicity (43).	MGB Eclipse	Population Hospital	0.712
Pine *et al* (11)	2006	African	133	255	All cases were histologically confirmed non-small cell primary tumors of the lungs. Patients were currently not taking antibiotics or steroid medications and had not previously undergone chemotherapy or radiation therapy (43).	Hospital controls were cancer-free patients recruited from the same hospital as the cases and were frequency matched to the cases by gender and ethnicity. Population controls were recruited from Baltimore City and the same counties to match cases by age, gender and ethnicity (43).	MGB Eclipse	Population Hospital	0.252
Zhang *et al* (18)	2006	Asian	1,106	1,420	Cases were newly diagnosed histopathologically confirmed lung cancer and patients were previously untreated with radio- or chemotherapy; no age, gender, tumor stage, or histology restrictions. Patients with previous cancer or metastasized cancer from other organs were excluded.	Cases had no individual history of cancer and the subjects were frequency-matched to the patients in terms of age (75 years) and gender.	ARMS-PCR	Population	0.721
Liu *et al* (21)	2008	Caucasian	1,787	1,360	Histologically confirmed lung cancer patients (≥ 18 years of age).	Controls were recruited among healthy friends, non-blood related family members of cancer patients, or spouses and friends of cardiothoracic surgery patients. Potential controls that carried a previous diagnosis of malignancy (other than nonmelanoma skin cancer) were excluded.	TaqMan	Hospital	0.615
Mittelstrass *et al* (22)	2008	Caucasian	633	1,294	Patients with an onset of disease at ≤50 years of age. Only newly diagnosed patients with histological or cytological confirmed primary lung cancer entered the study.	Matched by gender and according to 3-year age groups to the cases in a 1:2 matching design	MALDI-TOF	Population	0.454

HWE, Hardy-Weinberg equilibrium.

**Table II:** Frequency of the *MDM2* 309T>G polymorphism in the different populations.

			Cases			Controls		
Study (ref.)	Year	Ethnicity	TT	TG	GG	TT	TG	GG
Hu *et al* (10)	2006	Asian	166	373	178	274	538	271
Li *et al* (9)	2006	Caucasian	419	472	135	408	573	164
Lind *et al* (20)	2006	Caucasian	130	156	55	161	207	44
Park *et al* (17)	2006	Asian	113	280	189	122	299	161
Pine *et al* (11)	2006	Caucasian	150	167	54	182	187	52
Pine *et al* (11)	2006	African-American	111	20	2	203	47	5
Zhang *et al* (18)	2006	Asian	249	561	296	418	711	291
Liu *et al* (21)	2008	Caucasian	702	802	283	530	631	199
Mittelstrass *et al* (22)	2008	Caucasian	270	293	70	547	598	149

**Table III:** Frequency of the *MDM2* 309T>G polymorphism in the different subgroups.

	Cases			Controls			
Subgroup	TT	TG	GG	TT	TG	GG	Study (ref.)
Gender							
Females	191	230	63	215	294	78	Li *et al* (9)
Males	228	242	72	193	279	86	
Females	24	47	12	40	51	6	Lind *et al* (20)
Males	106	109	43	121	156	38	
Females	11	40	28	22	62	31	Park *et al* (17)
Males	19	62	45	100	237	130	
Females	346	381	139	302	362	91	Liu *et al* (21)
Males	350	424	147	230	272	103	
Females	95	103	30	197	232	50	Mittelstrass *et al* (22)
Males	175	190	40	350	366	99	
Histology							
Adenocarcinoma	196	231	76	408	573	164	Li *et al* (9)
Squamous cell carcinoma	98	98	28	408	573	164	
Adenocarcinoma	30	102	73	122	299	161	Park *et al* (17)
Squamous cell carcinoma	57	128	85	122	299	161	
Adenocarcinoma	82	171	108	418	711	291	Zhang *et al* (18)
Squamous cell carcinoma	113	241	122	418	711	291	
Adenocarcinoma	422	475	158	530	626	204	Liu *et al* (21)
Squamous cell carcinoma	178	186	59	530	626	204	
Adenocarcinoma	98	98	25	547	598	149	Mittelstrass *et al* (22)
Non-small carcinoma	382	415	130	408	573	164	Li *et al* (9)
Small cell carcinoma	31	37	3	408	573	164	
Non-small carcinoma	89	234	162	122	299	161	Park *et al* (17)
Small cell carcinoma	24	46	27	122	299	161	
Non-small carcinoma	183	205	50	547	598	149	Mittelstrass *et al* (22)
Small cell carcinoma	70	67	16	547	598	149	
Non-small carcinoma	130	156	55	161	207	44	Lind *et al* (20)
Non-small carcinoma	702	802	283	530	631	199	Liu *et al* (21)
Smoking status							
Ever	357	385	112	332	482	146	Li *et al* (9)
Never	62	87	23	76	91	18	
Ever	650	735	253	362	419	125	Liu *et al* (21)
Never	52	67	30	195	212	74	
Ever	195	206	46	165	173	40	Mittelstrass *et al* (22)
Never	71	77	21	369	413	105	
Ever	19	61	45	94	228	122	Park *et al* (17)
Never	11	41	28	28	71	39	
Ever	172	338	182	255	377	141	Zhang *et al* (18)
Never	77	223	114	193	334	150	

**Table IV:** Meta-analysis of the *MDM2* SNP309 T>G polymorphism in lung cancer.

Group	OR_AB/AA_ (95% CI)	OR_BB/AA_ (95% CI)	Genetic model	OR (95% CI) of assumed genetic model	Heterogeneity check	
					P-value	*I^2^*
Total	1.003 (0.931–1.081)	1.144 (1.037–1.262)	Recessive	1.143 (1.047–1.247)	0.056	47.20%
Caucasian	0.932 (0.850–1.022)	1.024 (0.896–1.170)	No association			
Asian	1.2 (1.050–1.372)	1.379 (1.142–1.665)	Dominant	1.260 (1.111–1.429)	0.145	48.30%
Gender						
Male	0.927 (0.817–1.053)	0.948 (0.796–1.131)	No association			
Female	0.939 (0.815–1.082)	1.239 (1.011–1.519)	Recessive	1.282 (1.062–1.548)	0.336	12.10%
Histology						
SCC	0.936 (0.779–1.124)	1.039 (0.796–1.358)	No association			
AC	0.991 (0.870–1.130)	1.204 (0.959–1.512)	No association			
SCLC	0.842 (0.653–1.086)	0.723 (0.501–1.043)	No association			
NSCLC	0.921 (0.838–1.014)	1.075 (0.944–1.225)	No association			
Smoking status						
Never	1.271 (1.063–1.521)	1.521 (1.214–1.905)	Dominant	1.328 (1.119–1.575)	0.151	40.50%
Ever	1.013 (0.846–1.212)	1.2 (0.886–1.625)	No association			

All ORs were derived from random-effects model if the heterogeneity was statistically significant, or else, from the fixed-effects model. Logistic regression analysis and heterogeneity check were performed using the same genetic model. AC, adenocarcinoma; SCC, squamous cell carcinoma; NSCLC, non-small carcinoma; SCLC, small cell carcinoma.

## References

[1] Jemal A, Bray F, Center MM, Ferlay J, Ward E, Forman D (2011). Global cancer statistics. CA Cancer J Clin.

[2] Chiang HC, Wang CY, Lee HL, Tsou TC (2011). Metabolic effects of CYP2A6 and CYP2A13 on 4-(methylnitrosamino)-1-(3-pyridyl)-1-butanone (NNK)-induced gene mutation - a mammalian cell-based mutagenesis approach. Toxicol Appl Pharmacol.

[3] Spitz MR, Wei Q, Dong Q, Amos CI, Wu X (2003). Genetic susceptibility to lung cancer: the role of DNA damage and repair. Cancer Epidemiol Biomarkers Prev.

[4] Wang LE, Cheng L, Spitz MR, Wei Q (2003). Fas A670G polymorphism, apoptotic capacity in lymphocyte cultures, and risk of lung cancer. Lung Cancer.

[5] Vonlanthen S, Heighway J, Kappeler A, Altermatt HJ, Borner MM, Betticher DC (2000). p21 is associated with cyclin D1, p16INK4a and pRb expression in resectable non-small cell lung cancer. Int J Oncol.

[6] Brooks CL, Gu W (2006). p53 ubiquitination: Mdm2 and beyond. Mol Cell.

[7] Alt JR, Bouska A, Fernandez MR, Cerny RL, Xiao H, Eischen CM (2005). Mdm2 binds to Nbs1 at sites of DNA damage and regulates double strand break repair. J Biol Chem.

[8] Bouska A, Lushnikova T, Plaza S, Eischen CM (2008). Mdm2 promotes genetic instability and transformation independent of p53. Mol Cell Biol.

[9] Li G, Zhai X, Zhang Z, Chamberlain RM, Spitz MR, Wei Q (2006). MDM2 gene promoter polymorphisms and risk of lung cancer: a case-control analysis. Carcinogenesis.

[10] Hu Z, Ma H, Lu D (2006). Genetic variants in the MDM2 promoter and lung cancer risk in a Chinese population. Int J Cancer.

[11] Pine SR, Mechanic LE, Bowman ED (2006). MDM2 SNP309 and SNP354 are not associated with lung cancer risk. Cancer Epidemiol Biomarkers Prev.

[12] Ma H, Hu Z, Zhai X (2006). Polymorphisms in the MDM2 promoter and risk of breast cancer: a case-control analysis in a Chinese population. Cancer Lett.

[13] Onat OE, Tez M, Ozcelik T, Toruner GA (2006). MDM2 T309G polymorphism is associated with bladder cancer. Anticancer Res.

[14] Ohmiya N, Taguchi A, Mabuchi N (2006). MDM2 promoter polymorphism is associated with both an increased susceptibility to gastric carcinoma and poor prognosis. J Clin Oncol.

[15] Alhopuro P, Ylisaukko-Oja SK, Koskinen WJ (2005). The MDM2 promoter polymorphism SNP309T-->G and the risk of uterine leiomyosarcoma, colorectal cancer, and squamous cell carcinoma of the head and neck. J Med Genet.

[16] Bond GL, Hu W, Bond EE (2004). A single nucleotide polymorphism in the MDM2 promoter attenuates the p53 tumor suppressor pathway and accelerates tumor formation in humans. Cell.

[17] Park SH, Choi JE, Kim EJ (2006). MDM2 309T>G polymorphism and risk of lung cancer in a Korean population. Lung Cancer.

[18] Zhang X, Miao X, Guo Y (2006). Genetic polymorphisms in cell cycle regulatory genes MDM2 and TP53 are associated with susceptibility to lung cancer. Hum Mutat.

[19] Chua HW, Ng D, Choo S (2010). Effect of MDM2 SNP309 and p53 codon 72 polymorphisms on lung cancer risk and survival among non-smoking Chinese women in Singapore. BMC Cancer.

[20] Lind H, Zienolddiny S, Ekstrom PO, Skaug V, Haugen A (2006). Association of a functional polymorphism in the promoter of the MDM2 gene with risk of nonsmall cell lung cancer. Int J Cancer.

[21] Liu G, Wheatley-Price P, Zhou W (2008). Genetic polymorphisms of MDM2, cumulative cigarette smoking and nonsmall cell lung cancer risk. Int J Cancer.

[22] Mittelstrass K, Sauter W, Rosenberger A (2008). Early onset lung cancer, cigarette smoking and the SNP309 of the murine double minute-2 (MDM2) gene. BMC Cancer.

[23] Bai J, Dai J, Yu H, Shen H, Chen F (2009). Cigarette smoking, MDM2 SNP309, gene–environment interactions, and lung cancer risk: a meta-analysis. J Toxicol Environ Health A.

[24] Gui XH, Qiu LX, Zhang HF (2009). MDM2 309 T/G polymorphism is associated with lung cancer risk among Asians. Eur J Cancer.

[25] Wan Y, Wu W, Yin Z, Guan P, Zhou B (2011). MDM2 SNP309, gene-gene interaction, and tumor susceptibility: an updated meta-analysis. BMC Cancer.

[26] Wo X, Han D, Sun H (2011). MDM2 SNP309 contributes to tumor susceptibility: a meta-analysis. J Genet Genomics.

[27] Bagos PG, Nikolopoulos GK (2007). A method for meta-analysis of case-control genetic association studies using logistic regression. Stat Appl Genet Mol Biol.

[28] Egger M, Davey Smith G, Schneider M, Minder C (1997). Bias in meta-analysis detected by a simple, graphical test. BMJ.

[29] Midgette AS, Wong JB, Beshansky JR, Porath A, Fleming C, Pauker SG (1994). Cost-effectiveness of streptokinase for acute myocar-dial infarction: A combined meta-analysis and decision analysis of the effects of infarct location and of likelihood of infarction. Med Decis Making.

[30] Higgins JP, Thompson SG, Deeks JJ, Altman DG (2003). Measuring inconsistency in meta-analyses. BMJ.

[31] Rose L SM, Cardwell CR, Jouvet P, McAuley DF, Blackwood B (2011). Automated versus non-automated weaning for reducing the duration of mechanical ventilation for critically ill adults and children. Cochrane Database Syst Rev.

[32] Higgins JPT, Green S (2011). Cochrane Handbook for Systematic Reviews of Interventions, Version 5.1.0, updated March 2011. The Cochrane Collaboration.

[33] Li H, Ha TC, Tai BC (2009). XRCC1 gene polymorphisms and breast cancer risk in different populations: a meta-analysis. Breast.

[34] Begg CB, Mazumdar M (1994). Operating characteristics of a rank correlation test for publication bias. Biometrics.

[35] Heist RS, Zhou W, Chirieac LR (2007). MDM2 polymorphism, survival, and histology in early-stage non-small-cell lung cancer. J Clin Oncol.

[36] Han JY, Lee GK, Jang DH, Lee SY, Lee JS (2008). Association of p53 codon 72 polymorphism and MDM2 SNP309 with clinical outcome of advanced nonsmall cell lung cancer. Cancer.

[37] Chien WP, Wong RH, Cheng YW, Chen CY, Lee H (2010). Associations of MDM2 SNP309, transcriptional activity, mRNA expression, and survival in stage I non-small-cell lung cancer patients with wild-type p53 tumors. Ann Surg Oncol.

[38] Dong J, Ren B, Hu Z (2011). MDM2 SNP309 contributes to non-small cell lung cancer survival in Chinese. Mol Carcinog.

[39] Jun HJ, Park SH, Lee WK (2007). Combined effects of p73 and MDM2 polymorphisms on the risk of lung cancer. Mol Carcinog.

[40] Wan JT (2008). Meta-analysis of association between SNP309 in MDM2 promoter and lung cancer susceptibility. Chin J Cancer Prev Treat.

[41] Higgins JP (2008). Commentary: Heterogeneity in meta-analysis should be expected and appropriately quantified. Int J Epidemiol.

[42] Groenwold RH, Rovers MM, Lubsen J, van der Heijden GJ (2010). Subgroup effects despite homogeneous heterogeneity test results. BMC Med Res Methodol.

